# Assessing the Recognition of Social Interactions Through Body Motion in the Routine Care of Patients with Post-Lingual Sensorineural Hearing Loss

**DOI:** 10.3390/jcm14051604

**Published:** 2025-02-27

**Authors:** Cordélia Fauvet, Léa Cantini, Aude-Eva Chaudoreille, Elisa Cancian, Barbara Bonnel, Chloé Sérignac, Alexandre Derreumaux, Philippe Robert, Nicolas Guevara, Auriane Gros, Valeria Manera

**Affiliations:** 1 CoBTeK Lab, Université Côte d’Azur, 06001 Nice, France; 2 UFR Medicine of Nice, Department of Speech Therapy, Université Côte d’Azur, 06001 Nice, France; 3 Institut Universitaire de la Face et du Cou (IUFC), Nice University Hospitals (CHU), 06100 Nice, France; 4 Institut Médico-Éducatif Cour de Venise 75, Association Autisme en IDF, 75003 Paris, France

**Keywords:** sensorineural hearing loss, biological motion, social interaction, point-light stimuli

## Abstract

**Background:** Body motion significantly contributes to understanding communicative and social interactions, especially when auditory information is impaired. The visual skills of people with hearing loss are often enhanced and compensate for some of the missing auditory information. In the present study, we investigated the recognition of social interactions by observing body motion in people with post-lingual sensorineural hearing loss (SNHL). **Methods:** In total, 38 participants with post-lingual SNHL and 38 matched normally hearing individuals (NHIs) were presented with point-light stimuli of two agents who were either engaged in a communicative interaction or acting independently. They were asked to classify the actions as communicative vs. independent and to select the correct action description. **Results:** No significant differences were found between the participants with SNHL and the NHIs when classifying the actions. However, the participants with SNHL showed significantly lower performance compared with the NHIs in the description task due to a higher tendency to misinterpret communicative stimuli. In addition, acquired SNHL was associated with a significantly higher number of errors, with a tendency to over-interpret independent stimuli as communicative and to misinterpret communicative actions. **Conclusions:** The findings of this study suggest a misinterpretation of visual understanding of social interactions in individuals with SNHL and over-interpretation of communicative intentions in SNHL acquired later in life.

## 1. Introduction

Effective communication is fundamental to human interaction: one individual uses verbal and non-verbal information perception processes to interpret the mental states of another individual and deduce his/her intentions based on subtle sensory cues. However, hearing loss (HL), which refers to a partial or total inability to hear sounds in one or both ears, suppresses the auditory information that contributes to social interaction understanding and induces a need to rely more consistently on visual information to infer communicative intentions and understand the spoken message [1]. Indeed, static components (such as interpersonal distance, body orientation, physical contact and posture) and dynamic components (such as eye-gaze, facial expressions, gestures and interpersonal synchrony) can inform observers about the intentions of agents, the type of relationship and their emotional state [2,3,4,5,6]. The spoken message is enhanced, contextualized, clarified and supported by this visual information, which proves essential in cases of HL [1].

**Figure 1:**
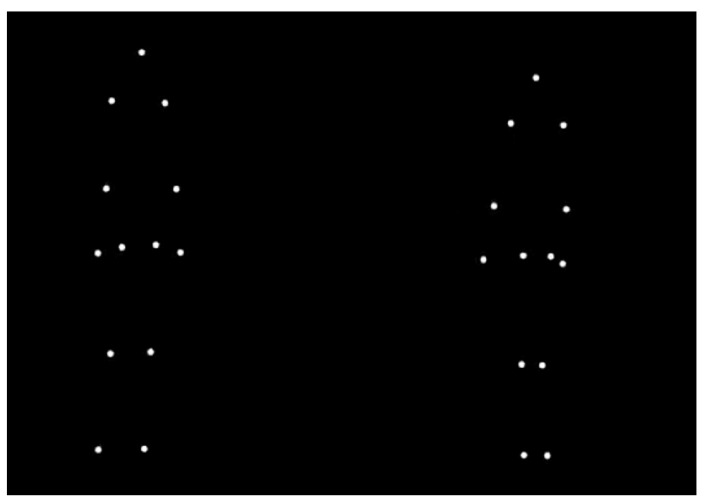
Example of two-agent point-light stimulus.

Body motion (BM) is a rich source of information for interpreting social interactions [7]. Using point-light displays, in which the movements of a body are represented by a small number of point lights indicating the major joints of a moving person (see Figure 1), it has been shown that the information available in BM is sufficient not only for the recognition of the actions of single individuals [8,9] but also for the recognition of the communicative intentions [10,11,12] and the emotional states [13] of interacting agents. Thus, the ability to recognize BM provides crucial cues for people with HL, enabling them to interpret their environment and understand the intentions of others to respond appropriately with adaptative social behavior, which is invaluable for daily activities [7,14]. In fact, studies have shown that patients with impaired BM processing also show deficits in social cognition in everyday life and vice versa [7].

HL is typically categorized into three main types: conductive HL, sensorineural HL (SNHL) and mixed HL. This study focuses on SNHL, which is the most common and permanent type of HL. It involves damage to the inner ear or the auditory nerve, which transmits sound signals to the brain. It has been discussed multiple times that HL has a strong impact on the quality of life and emotional states of patients by initiating a cascade of psychological and social challenges [15,16]. Due to their sensory impairment, individuals with HL may avoid socially demanding situations, leading to isolation, which can frequently evolve into irritability and reduced self-esteem [15]. As these psychological symptoms intensify, they further impact social behavior, creating a self-perpetuating cycle, especially in acquired HL. In addition, SNHL is often associated with tinnitus (“the perception of sound without an external source” [17]), which can significantly affect daily functioning and reduce quality of life by up to 5% [17]. Studies have shown that individuals with hearing impairment are more vulnerable to depression than the general population, with some studies indicating clinical depression rates even 4.8 times higher [18]. Critically, the tendency to withdraw from social interactions does not just affect mental health; it can significantly impair emotional understanding and social cognition [19,20], with the resulting maladaptive communication strategies causing individuals to perceive their social skills as inadequate [15].

Yet an increasing number of studies suggest that sensory loss leads to the enhancement of the other intact sensory modalities due to the phenomenon of cross-modal plasticity [21,22,23]. Indeed, auditory deprivation can induce the structural and functional reorganization of the cortical architecture in the visual cortex [24], with the auditory regions becoming more sensitive to vision quite rapidly [25,26,27,28]. It has been described as the “sensory compensation hypothesis”: the brain areas associated with the impaired sensory modality may adapt to process inputs from one or more of the remaining intact sensory systems or, alternatively, develop enhanced functional and processing abilities [29]. Therefore, the visual cross-modal activity of the auditory cortex is often described as compensatory, indicating that individuals with HL depend more heavily on their intact visual systems to perceive and interpret their environment compared with normally hearing individuals (NHIs) [14]. For instance, compared with NHIs, people with HL show advantages in visual localization [21,30,31], peripheral position discrimination [24], visuomotor synchronization [32], motion processing [33] and motion detection [31,34,35], especially for stimuli presented in the visual periphery [24,30,33,36,37,38,39].

The ability to recognize information directly relevant to social interactions is less studied, but there is some evidence that the performance of people with HL is comparable with—or even better than—that of NHIs. For instance, neural processing of visual information relevant to communication (postures, mimics, gestures, gaze) is faster in people with post-lingual HL [40]. However, contradictory results are sometimes found in the literature, particularly for the question of facial emotion recognition. According to some, individuals with HL show better discrimination of emotional expressions and local facial features [41,42] and are faster at emotional facial expression identification [43]. However, in a population of cochlear-implanted patients, Ambert-Dahan and her team showed that acquired and progressive HL are associated with a reduction in emotional sensitivity to visual stimuli [44]. Their study analyzed these results in relation to the issue of lip-reading, a common and critical compensation mechanism in HL [44,45]. According to them, “progressive HL forces patients to focus on speech-relevant facial cues, and this could prevent them from paying attention to not only cues coming from the upper part of the face, such as eye movements, but also perhaps to non-linguistic cues coming from the mouth” and thus “to focus more on verbal than non-verbal cues, and pay less attention to non-verbal information” [44]. Two recent studies focused on BM perception. A study by Quant and colleagues [46] showed that individuals with early HL (term used by Quant and colleagues, to be understood as pre-lingual) reported significantly less effort in recognizing single-agent biological-motion stimuli and scrambled motion depicted in point-light displays compared with NHIs. A study by Simon and colleagues [14] explored the cortical processing of single-agent biological-motion stimuli that conveyed either non-communicative or communicative information in individuals with early HL and NHIs. The individuals with early HL were faster at classifying the communicative gestures relative to the NHIs. Both studies focused on individuals with pre-lingual congenital HL and analyzed the ability to correctly classify single-agent stimuli (individual actions vs. scrambled motion [46] or communicative gestures vs. non communicative gestures vs. scrambled motion [14]) without analyzing the precise action description provided by the participants. As pre- and post-lingual HL occur at different times during development, they require separate investigations to understand their mechanisms and impact on communications. Several studies have investigated differences in brain maturation and cross-modal reorganization in children with SNHL, showing differences in performance in integrating auditory–visual information depending on whether the children had received adequate speech stimulation, with a lack of acquired age-appropriate spoken language skills being associated with a tendency to rely more on visual information when faced with conflicting auditory and visual cues [47]. However, the literature on the possible behavioral or cognitive enhancements experienced by patients with HL is characterized by results that are both heterogenous and inconsistent. Lastly, as said in the recommendations from the French Society of Otorhinolaryngology and Head and Neck Surgery, the French Society of Audiology and the French Society of Geriatrics and Gerontology, “it is recommended that cognitive, verbal, non-verbal, emotional, and lip-reading skills are assessed as part of the speech-language pathology assessment to optimize management” of hearing impairment [16].

So, with over 430 million people worldwide suffering from disabling HL [48] and relying on their remaining senses to maintain social interactions in their daily activities, it is crucial to better understand the mechanisms underlying the understanding of communicative interactions with hearing loss. The aim of the present study is therefore to investigate the recognition of social interactions by observing body motion in people with post-lingual HL. As the phenomenon of cross-modal plasticity is also observed in post-lingually acquired HL [49], we may hypothesize an advantage of individuals with HL in recognizing communicative interactions from body motion compared with NHIs.

## 2. Materials and Methods

### 2.1. Participants

This study was approved by the Comité d’Éthique pour les Recherches Non Interventionnelles de l’Université Côte d’Azur (CERNI) AVIS n° 2020-62 on 2 July 2020. All participants signed an informed consent form before the start of this study. Participants with bilateral severe-to-profound sensorineural hearing loss (SNHL) were recruited at an academic tertiary care center during a pre-cochlear implant assessment. The severity of the HL was tested using the Pure Tone Average loss, which is calculated by averaging hearing thresholds at four specific frequencies: 500 Hz, 1000 Hz, 2000 Hz and 4000 Hz. Speech perception in noise (cocktail party) was also assessed using the Test Vocal Rapide dans le Bruit [50], without lip-reading and with a gradually decreasing speech-to-noise ratio. The NHIs were recruited through an online survey. Participants with self-reported hearing impairment or cognitive impairment (as assessed through standard verbal and non-verbal memory tests, such as the Grober and Buschke Test [51] and Doors and People Test [52]) were not included. All participants had normal or corrected-to-normal vision.

### 2.2. Materials and Procedure

As observed social interactions imply the presence of at least two persons, we selected point-light stimuli of two agents that were either engaged in a communicative interaction (e.g., agent A asks agent B to squat down, and agent B squats down) or acting independently (agent A turns over, agent B squats down), selected from an existing database [11]. We asked the participants both to classify the stimuli as communicative interactions vs. independent actions and to provide the correct action description. The stimuli for the point-light task were selected from the Multilingual CID-5 database [11,53]. They consisted of ten videos with a black background where two agents perform some actions. Each agent is represented by 13 light points indicating the head, shoulders, elbows, wrists, hips, knees and feet. Ten stimuli were selected: five communicative (in which agent A performs a communicative gesture toward agent B, who responds accordingly) and five independent (in which agents A and B perform independent actions). The following stimuli were selected: “Imitate me”, “Look at the ground”, “Stand up”, “Pick this up” and “Move this down” for the communicative conditions and “Sneeze”, “Drink”, “Stretch”, “Lateral steps” and “Jump” for the independent conditions (see [53] for a complete description of the stimuli). We selected stimuli that were correctly recognized by at least 70% of healthy participants in a free-description format [10]. Each video lasted between 4 and 11 s and was followed by two single-choice questions. First, the participants were asked to classify the actions as communicative vs. independent (Task A, “interaction score”). Second, the participants were asked to choose the correct action description among 5 alternatives, presented in French (Task B, “description score” [11]). The alternatives included the correct description, two incorrect communicative alternatives and two incorrect independent alternatives. The questions were presented on the screen until answered, with no time restriction (reaction times were not measured). Each video was shown once. The videos were viewed in the same order by all participants. The participants received no feedback regarding response accuracy.

### 2.3. Data Analysis

The distribution of the data (numbers of correct responses to interaction and description scores and numbers of mistakes) for the participants with hearing loss and controls did not follow a normal distribution, as demonstrated by the Shapiro–Wilk test (*p* < 0.05). We therefore employed non-parametric tests. RStudio 4.3.2 software was used. We performed the Wilcoxon rank-sum test to compare the number of correct answers for tasks A and B between the participants with SNHL and the NHIs. We performed the same to compare the types of errors in tasks A and B and to compare the scores between the individuals with congenital or acquired SNHL. Significant results are reported as *p* < 0.05 (*p* < 0.05 “*”, *p* < 0.01 “**”, *p* < 0.001 “***”).

## 3. Results

### 3.1. Participants

In total, 76 participants were enrolled, including a group of subjects with post-lingual SNHL (*n* = 38) and a group of NHIs matched by sex, age and education level (*n* = 38). Table 1 summarizes the participants’ demographic information. All SNHL participants had severe to profound HL of various etiologies (congenital (*n* = 8), Ménière’s disease (*n* = 7), age-related (*n* = 3), sudden idiopathic (*n* = 2), traumatic (*n* = 2), others (*n* = 1), unknown (*n* = 15)), and each was presumed to have acquired age-appropriate spoken language abilities. Based on the type of onset of hearing loss, the SNHL participants were divided into two groups: a “congenital SNHL” group (*n* = 8) and an “acquired SNHL” group (*n* = 15) (Table 2).

### 3.2. Results, Task A

The total score for the interaction task in the individuals with SNHL (Mdn = 7) did not differ significantly from the NHIs (Mdn = 8) (W = 820, *p* = 0.299). By dividing the errors into communicative ones (errors on communicative stimuli) and independent ones (errors on independent stimuli), we analyzed the overall types of errors. The number of communicative errors in the individuals with SNHL (Mdn = 0) did not differ significantly from the NHIs (Mdn = 1) (W = 743.5, *p* = 0.811). The number of independent errors in the individuals with SNHL (Mdn = 2) did not differ significantly from the NHIs (Mdn = 1.5) (W = 655, *p* = 0.478).

### 3.3. Results, Task B

**Figure 2:**
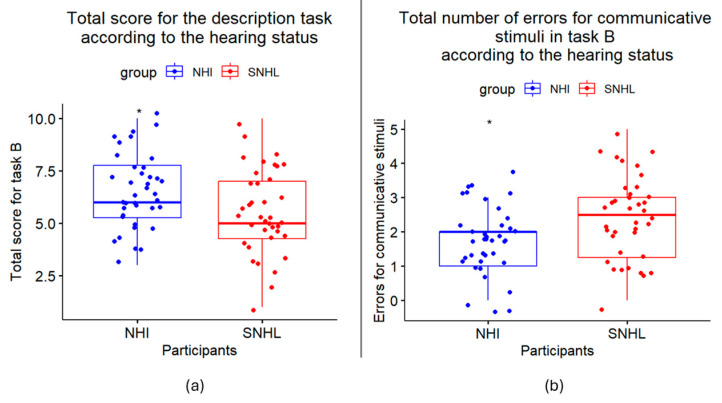
(**a**) Boxplot comparison of total description task (Task B) scores between the normally hearing individual (NHIs) and sensorineural hearing loss (SNHL) groups using the Wilcoxon rank-sum test and (**b**) boxplot comparison of total number of errors for communicative stimuli between the NHIs and SNHL groups using the Wilcoxon rank-sum test. Significant results are reported as *p* < 0.05 *.

For the description task, the individuals with SNHL (Mdn = 5) performed significantly lower than the NHIs (Mdn = 6) (W = 925.5, *p* = 0.033 *), as shown in Figure 2a. To further explore this result, we analyzed the overall types of errors for Task A. The errors were divided into communicative ones (errors on communicative stimuli) and independent ones (errors on independent stimuli). The participants with SNHL (Mdn = 2.5) made significantly more errors than the NHIs (Mdn = 2) when the stimuli were communicative (W = 489, *p* = 0.012 *) but not when the stimuli were independent (Mdn = 2, W = 617.5, *p* = 0.266) (Figure 2b). To understand why more errors were made for the participants with SNHL for the communicative stimuli, under-interpretation (when the participants chose independent responses instead of communicative ones) and misinterpretation (when the participants chose communicative responses but not the correct ones) were distinguished. A significant difference was found due to misinterpretation: the participants with SNHL tended to choose incorrect communicative responses more often than the NHIs (Mdn = 1, W = 467, *p* = 0.005 **).

### 3.4. Exploration of Differences in HL

**Figure 3:**
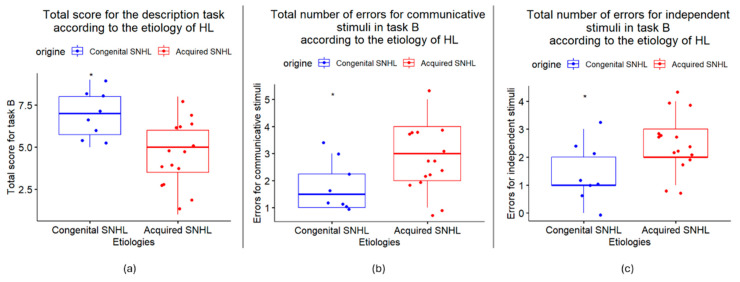
(**a**) Boxplot comparison of total description task (Task B) scores between the congenital sensorineural hearing loss (congenital SNHL) and acquired sensorineural hearing loss (acquired SNHL) groups using the Wilcoxon rank-sum test; (**b**) boxplot comparison of total number of errors for communicative stimuli between the congenital SNHL and acquired SNHL groups using the Wilcoxon rank-sum test and (**c**) boxplot comparison of total number of errors for independent stimuli between the congenital SNHL and acquired SNHL groups using the Wilcoxon rank-sum test. Significant results are reported as *p* < 0.05 *.

The total score for the interaction task in the individuals with congenital SNHL (Mdn = 8.5) did not differ significantly from the individuals with acquired SNHL (Mdn = 7) (W = 90, *p* = 0.051). However, for the description task, the individuals with congenital SNHL (Mdn = 7) performed significantly better than the individuals with acquired SNHL (Mdn = 5) (W = 99.5, *p* = 0.011 *) (Figure 3a). Moreover, the participants with congenital SNHL (Mdn = 1.5) made significantly fewer errors than the participants with acquired SNHL (Mdn = 3) when the response was communicative (W = 30, *p* = 0.049 *), as well as when the response was independent (Mdn = 1/2, W = 24, *p* = 0.017 *) (Figure 3b,c). To understand why more errors were made for the participants with acquired SNHL for the communicative stimuli, under-interpretation (when the participants chose independent responses instead of communicative ones) and misinterpretation (when the participants chose communicative responses but not the correct ones) were distinguished. For the independent stimuli, over-interpretation (when the participants chose communicative responses instead of independent ones) and misinterpretation (when the participants chose independent responses but not the correct ones) were distinguished. A significant difference was found due to over-interpretation—the participants with acquired SNHL tended to overattribute communicative intentions to independent interaction (Mdn = 2) compared with the individuals with congenital SNHL (Mdn = 0.5) (W = 16.5, *p* = 0.004 **)—and to misinterpretation of communicative stimuli: the participants with acquired SNHL tended to choose more incorrect communicative responses (Mdn = 1.6) compared with the individuals with congenital SNHL (Mdn = 0.75) (W = 29.5, *p* = 0.038 *).

## 4. Discussion

HL suppresses the auditory information that contributes to social interaction understanding and induces a greater reliance on visual information [1]. Brain plasticity generates behavioral specificities in individuals with HL, with improved performance in several visual and visuomotor skills [14,24,43,54]. Recently, it has been shown that compared with NHIs, individuals with early HL are faster and more confident in recognition of individual [46] and communicative [14] actions from point-light stimuli, which suggests faculties in inferring socially relevant information from observing body movements.

In the present study, our goal was to investigate this ability to recognize communicative and individual actions from point-light stimuli in individuals with post-lingual SNHL, tested in the context of a pre-cochlear implantation assessment. Specifically, participants with post-lingual SNHL and NHIs were presented with point-light stimuli of two agents, asked to classify their actions as communicative or independent (Task A—interaction score) and to select the correct description from five alternatives (Task B—description score). As it has been shown that cross-modal plasticity may be induced by auditory deprivation itself, even in late-onset SNHL, regardless of the duration of auditory deprivation [55,56], we hypothesized that the participants with post-lingual SNHL would show improved performance in our task compared with the age-, sex- and education-matched NHIs.

Contrary to our expectations, our results showed that the classifying scores were slightly lower in the participants with SNHL compared with the matched NHIs and that the description scores were significantly lower in the participants with SNHL compared with the matched NHIs. This lower performance was specifically due to a higher number of mistakes in the communicative stimuli, while the performance in recognizing the independent interactions was similar in the two groups. In addition, the performance of the individuals with acquired SNHL was even lower than that of the individuals with congenital SNHL due to a higher number of mistakes in communicative stimuli and a tendency to over-interpret independent stimuli as communicative. These results may suggest that cross-modal reorganization in post-lingual SNHL could be less important compared with pre-lingual SNHL [53] and that the earlier the onset of hearing loss, the better the performance in visual integration. Moreover, it questions the existence of a maladaptive plasticity where the brain reorganization resulting from late-onset SNHL would ultimately have a negative impact on non-verbal communication skills and therefore on the quality of life of individuals with HL. As a lack of verbal information makes others’ intentions to engage in communicative interactions harder to detect, individuals with post-lingual acquired SNHL would develop the tendency to over-interpret actions as communicative so as not to lose opportunities of social interactions. Further studies should corroborate this finding using a bigger variety of communicative and individual actions and explore links with participants’ mentalization ability.

Differences in methodology may contribute to explaining why we found results partially different from those of previous studies on early HL [14,46]. First, those studies employed single-agent point-light action stimuli, which may be easier to recognize compared to two-agent interactions. Indeed, understanding the actions of two agents requires a split of the observer’s attentional resources to decode different actions and detect the presence or absence of interpersonal synchrony (e.g., in a communicative interaction, the action of the communicative agent precedes the action of the respondent and the two actions must be well-synchronized in space and time). In the present study, the choice was made to include two-agent interactions to be as representative and close as possible to the reality of the social interactions and daily life of the participants. It is possible that, employing our stimuli, the participants with early HL would have performed similarly to our subjects, with lower performance in recognizing two-person independent actions compared with the NHIs. Second, due to the constraints of EEG and fMRI techniques, the previous studies investigated participants’ ability to classify actions without analyzing the precise action descriptions [14,46]. Using these classification paradigms, the advantages for the HL participants were found in response latencies and in self-confidence in providing a correct response, while behavioral performance was comparable in the participants with HL and NHIs. In our study, behavioral performance was comparable in the participants with SNHL and NHIs, but no measures were taken for rapidity and self-confidence. Further studies should clarify if the facilitation in processing biological-motion stimuli in early HL translates into improved performance in correctly describing and interpreting observed actions or not or if reaction time and self-confidence also differ between post-lingual SNHL and NHIs. Thus, before concluding that late-onset and early SNHL result in different performances in biological-motion recognition tasks, it would be important to compare the participants in the two groups using the very same experimental setup.

Furthermore, the links between hearing loss and cognitive decline are increasingly being explored, and in July 2024, the Lancet International Commission identified HL as the largest potentially modifiable risk factor for dementia in midlife, alongside other health and lifestyle factors, such as depression and social isolation [57]. Indeed, one of the causal hypotheses behind the association between HL and the increased risk of dementia is the occurrence of psychosocial factors such as social isolation (real or perceived), loneliness, apathy, increased negativity and depression [57,58,59]. However, Livingston and her team have shown that treating hearing loss reduces the risk of dementia by 7%, reducing social isolation reduces the risk of dementia by 5%, and effectively treating depression reduces the risk of dementia by 3% [57]. Thus, this work, which questions the misinterpretation of communicative intentions in a population of late-onset HL individuals, opens up new perspectives for managing social isolation and reducing the risk of dementia in the presence of HL. Another well-known causal hypothesis is “the cognitive-load hypothesis” [59]. According to this idea, HL causes a reallocation of cognitive resources to compensate for the lack of information perceived in the environment, “eventually resulting in cognitive reserve depletion” [59]. Excessive cognitive effort devoted to auditory perceptual processing can lead to structural changes in the brain and neurodegeneration, ultimately impairing other cognitive functions, leading to cognitive decline [59]. It may therefore be interesting to repeat this experiment, also looking for possible correlations between the performance and cognitive scores, in order to detect possible cognitive inequalities between the NHIs and people with HL.

Despite the preliminary results being of interest in the context of post-lingual hearing loss, some limitations of our study can be mentioned. First, this study was performed on a relatively small sample of participants. It would be important to repeat this study with a bigger sample size to allow exploring of the effects of variables such as the precise duration of the hearing loss and the severity of the HL, factors that may significantly affect cross-modal plasticity [52]. The differences between congenital and acquired HL must be qualified, as there were marked differences between the populations in sex or, more specifically, in age, which has been found to be significantly correlated with performance. For example, some authors have shown that older adults have performed significantly more poorly than younger adults at lip-reading or integrating visual stimuli in a degraded state [60,61]. It would therefore be interesting to determine whether these differences in performance could also be related to age at the onset of HL or the duration of the HL rather than just the type of onset. Second, in our study, we employed a low number of point-light stimuli. As the task was administered in a clinical setting, it was indispensable to employ a short test battery. It would be important to employ a bigger variety of biological-motion stimuli, including single-agent communicative and individual actions, to compare our results with those of previous studies on congenital hearing loss. Third, as previous results have shown that the rapidity of the response and the rater confidence, rather than the response accuracy, differentiate participants with hearing loss and healthy controls, it would have been important to assess these indexes.

## 5. Conclusions

In conclusion, the present results confirm no significant differences between the participants with SNHL and the matched NHIs in the classification of communicative vs. independent actions. However, the individuals with SNHL performed significantly poorer than the NHIs in describing the communicative stimuli. Important inter-individual variability was also observed in the task performance of the participants with SNHL, with an effect of etiology. Thus, the present results are a further reminder of the importance of providing personalized and individualized care, considering both the cognitive and sensory profiles of patients.

## Figures and Tables

**Table 1:** Demographic and clinical characteristics of the participants. SNHL: sensorineural hearing loss; NHIs: normally hearing individuals; IQR: interquartile range.

		SNHL (*n* = 38)	NHIs (*n* = 38)	Total (*N* = 76)
Sex	Male	12	12	24
	Female	26	26	52
Age	Median (IQR)	56.5 (30)	57 (28.75)	56.5 (30.5)
	Q1–Q3	39.5–69.5	41–69.75	39.5–70
	Min.–Max.	19–82	20–82	19–82
Education	Primary	8	4	12
	Secondary	8	7	15
	Higher	22	27	49

**Table 2:** Demographic and clinical characteristics of participants with congenital and acquired sensorineural hearing loss. IQR: interquartile range.

		Congenital (*n* = 8)	Acquired (*n* = 15)
Sex	Male	1	7
	Female	7	8
Age	Median (IQR)	30 (10)	67 (19.5)
	Q1–Q3	28–38	54.5–74
	Min.–Max.	19–52	38–82
Education	Primary	1	3
	Secondary	3	0
	Higher	4	12

## Data Availability

Anonymized data will be shared for research purposes upon request.
